# Knowledge, Attitude, Practice, and Barriers Toward Pharmacovigilance Among Pharmaceutical Sales and Marketing Personnel in Saudi Arabia: A Cross-Sectional Study

**DOI:** 10.3390/pharmacy13050145

**Published:** 2025-10-09

**Authors:** Muath A. Alsalloum, Mohammed A. Almutairi, Saud M. Alsahali, Waleed M. Altowayan

**Affiliations:** 1 Department of Pharmacy Practice, College of Pharmacy, Qassim University, Buraidah 51452, Saudi Arabia; s.alsahali@qu.edu.sa (S.M.A.); w.altowayan@qu.edu.sa (W.M.A.); 2 Pharmacovigilance Team, MS Pharma, Riyadh 13315, Saudi Arabia; ph.mohammed.am@gmail.com

**Keywords:** medication safety, pharmacovigilance, adverse drug reaction, pharmaceutical sales, pharmaceutical marketing, KAP, barriers, Food and Drug Administration, questionnaire

## Abstract

Sales and marketing personnel are among the most knowledgeable individuals regarding the safety of the medications they promote. No previous work has assessed pharmaceutical sales and marketing personnel’s knowledge, attitude, practice (KAP), and barriers toward pharmacovigilance (PV) in Saudi Arabia; therefore, the present study aimed to assess these aspects and to scrutinize their associations with the subjects’ baseline characteristics. A validated questionnaire comprising five sections (baseline characteristics, knowledge, attitude, practice, and barriers) was disseminated via email networks and social media platforms between 18 March and 31 May 2025. All employees working in the sales and marketing departments of pharmaceutical companies in Saudi Arabia were eligible to participate. Participants’ responses were categorized as good or poor knowledge, positive or negative attitude, good or poor practice, and challenging or non-challenging work environment, based on the cumulative score in each respective section, using a 60% cutoff. A total of 400 participants completed the survey. Of these, about one-third (37.3%) had 2–4 years of professional experience and two-thirds (63%) were employed by multinational companies. Overall, 57% and 83.5% had good knowledge and positive attitude, respectively. The work environment was considered non-challenging by 92.8% of participants, and 61% reported good practice. We noted that holding a non-pharmacy degree was a significant predictor of poor knowledge and a challenging work environment. Additionally, employment in a local company was significantly associated with poor knowledge and practice. Pharmaceutical sales and marketing personnel in Saudi Arabia demonstrated acceptable levels of KAP and reported few barriers toward PV, with an opportunity for improvement.

## 1. Introduction

Pharmacovigilance (PV) is defined by the WHO as “the science and activities relating to the detection, assessment, understanding and prevention of adverse effects or any other medicine/vaccine related problem [1].” Adverse effects and drug-related problems impose a significant burden on the healthcare system by increasing the risk of patient injury, high healthcare costs, hospitalization, prolonged length of hospital stays, readmission, morbidity, and mortality [2,3,4,5,6,7,8,9]. In Saudi Arabia, pharmaceutical sales and marketing personnel are legally required to be licensed pharmacists. They receive intensive product-specific training, which makes them well-informed about the medications they promote. Therefore, their knowledge, attitude, practice (KAP), and barriers toward PV are of paramount importance.

According to the Saudi Food and Drug Authority (SFDA) guideline on Good Pharmacovigilance Practices (GVP), all staff who interact with healthcare professionals (HCPs) are part of the PV system, including sales and marketing employees [10]. Marketing authorization holders (MAHs) are responsible for ensuring that an appropriate infrastructure that supports good PV practices is in place, including a well-structured PV system, adequate training, as well as a blame-free culture [10]. MAHs must have a full-time qualified person responsible for pharmacovigilance (QPPV) who resides in Saudi Arabia, who is expected to create the local standard operating procedures (SOPs) for handling PV activities, e.g., handling of local individual case safety reports (ICSRs), preparing and updating pharmacovigilance system master file (PSMF) and pharmacovigilance sub-system file (PSSF), as well as preparing and submitting periodic safety update report/periodic benefit-risk evaluation report (PSUR/PBRER) [10]. QPPV should facilitate employees’ compliance with the PV system by ensuring easy access to PV tools like forms and contacts as well as by monitoring reporting compliance via key-performance indicators (KPIs) and field audits [10].

Most existing literature focuses on KAP toward PV among HCPs in settings like hospitals and community pharmacies. For instance, Abdulsalim and colleagues surveyed 209 community pharmacists in Qassim, Saudi Arabia, and reported good knowledge and a highly positive attitude; however, the majority of respondents (85.6%) did not know how to report adverse drug reactions (ADRs) [11]. Similarly, another cross-sectional study by Jose et al. in Oman included 107 community pharmacists and reported a lack of awareness on how to report ADRs among the top discouraging factors among participants [12]. Additionally, 377 hospital and community pharmacists in Texas, the US, were surveyed and 70% reported not having ever reported an ADR [13].

Pharmaceutical sales and marketing personnel’s KAP and barriers toward PV remain unassessed either locally or globally. Hence, the primary objective of this study was to assess pharmaceutical sales and marketing personnel’s KAP and barriers toward PV in Saudi Arabia and to scrutinize their associations with the subjects’ baseline characteristics.

## 2. Materials and Methods

### 2.1. Study Design and Participants

This study employed a cross-sectional design and utilized a self-administered online survey, which was developed based on an extensive review of the literature on PV KAP studies, with adaptation to the local context of pharmaceutical sales and marketing personnel. It was further refined in the validation process to ensure clarity and relevance. It was validated for its structure, face, and content, and tested through a pilot study. Validation was conducted by a panel of five experts, including three PV specialists and two academic researchers with expertise in survey methodology. The pilot sample comprised 25 pharmaceutical sales and marketing personnel in Saudi Arabia. Feedback indicated no difficulties, and thus no changes were required beyond the initial modifications recommended by the validation panel. The questionnaire was hosted on Google Forms and disseminated through email networks as well as social media platforms [i.e., LinkedIn (version 9.31.5267), X (version 11.25.1), and WhatsApp (version 2.25.26.72)] to maximize reach among pharmaceutical sales and marketing personnel across Saudi Arabia between 18 March and 31 May 2025. The present cross-sectional study aligns with the Strengthening the Reporting of Observational Studies in Epidemiology (STROBE) guidelines to ensure methodological robustness.

### 2.2. Sample Size and Sampling Technique

The present study employed a convenience sampling approach, and all employees working in the sales and marketing departments of pharmaceutical companies in Saudi Arabia were eligible to participate. Using the Raosoft sample size calculator, we factored in a population size of 20,000 (as official figures for pharmaceutical sales and marketing personnel are not available), a 95% confidence level, 5% margin of error, and an expected response rate of 50%. A minimum sample size of 377 was calculated [14].

### 2.3. Survey Design

The questionnaire was divided into five main sections designed to collect data on demographics and work-related characteristics, knowledge, attitude, practice, and barriers toward PV. The measurement of the four aspects toward PV was conducted using nine items for knowledge, four items for attitude, eight items for practice, and 18 items for barriers. Participants’ responses were categorized as good or poor knowledge, positive or negative attitude, good or poor practice, and challenging or non-challenging work environment, based on the cumulative score in each respective section, using a 60% cutoff [15]. For instance, those who answered six items or more correctly in the knowledge section were considered to have good knowledge, and those who identified 11 items or more in the barriers section as factors hindering them from being compliant to the PV system were considered to have a challenging work environment. The questionnaire was pretested on a pilot sample to ensure clarity and is available in the Supplementary File S1. Participants were required to answer all questions prior to submitting the questionnaire in order to ensure complete responses and avoid missing data.

### 2.4. Statistical Analysis

Data were analyzed using SPSS Statistics, version 26. Descriptive and inferential statistical analyses were performed. Associations between dependent variables, i.e., KAP and barriers and independent variables, i.e., current position (sales vs. marketing department), region (central vs. non-central), academic qualification (pharmacy vs. non-pharmacy degree), years of experience (<4 vs. ≥4), type of company (local vs. multinational), and frequency of interaction with HCPs (daily vs. non-daily) were examined using the chi-square test and the equivalent Fisher’s exact test. These independent variables were selected after discussions among the authors, primarily because they were expected to provide useful insights for decision makers aiming to target specific subgroups with the necessary interventions.

### 2.5. Ethical Considerations

The study protocol was reviewed and approved by the Committee of Research Ethics at Qassim University (No. 25-30-11). Participation was voluntary, and respondents were permitted to withdraw at any time without providing a reason. Anonymity and confidentiality were maintained, with no identifying information collected.

## 3. Results

### 3.1. Baseline Characteristics

A total of 400 participants completed the survey. Of these, the majority (94.3%) and (92.5%) were working in a sales department and aged between 25 and 34, respectively. About one-third (37.3%) had 2–4 years of professional experience and two-thirds (63%) were employed by multinational companies. Most of the respondents (97.8%) and (86%) hold a degree in pharmacy and were interacting with HCPs on a daily or almost daily basis, respectively. Table 1 summarizes the participants’ baseline characteristics.

### 3.2. Knowledge

Overall, 57% of respondents demonstrated good knowledge. However, over half (56%) were unable to identify the correct definition of PV, and almost half (51.2%) did not know the primary purpose of PV activities. Table 2 illustrates the respondents’ answers to the knowledge section.

### 3.3. Attitude

Although only 51.8% of participants deemed the ADRs reporting form not complex to fill out, the majority of respondents (83.5%) manifested a positive attitude. Further details regarding attitude are highlighted in Table 3.

### 3.4. Practice

Overall, 61% of participants reported good practice. Notably, more than one-third of respondents (39.8%) reported not having ever reported an ADR, and more than half (59.3%) reported not having reported an ADR in the last 12 months. One-third (33.2%) indicated that they do not report the ADR within one business day, with about one-fifth (19%) having no specific time frame. Table 4 highlights the participants’ answers to the practice section.

### 3.5. Barriers

**Figure 1:**
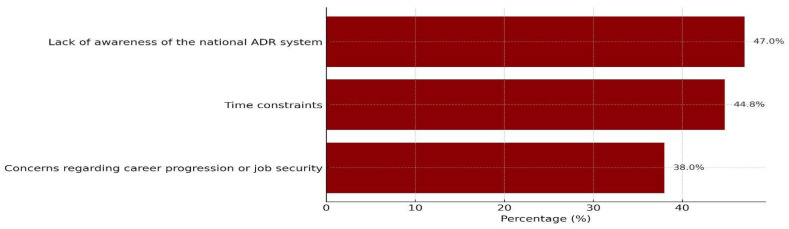
The most commonly perceived barriers to ADR reporting.

Although 92.8% of respondents reported having non-challenging work environments, nearly half (44.8%) and (47%) felt they lack sufficient time to complete ADR reports and were not aware of the national ADR reporting system and how to use it, respectively. Additionally, over one-third (38%) expressed concern that reporting ADRs could negatively impact their career progression or job security. The most commonly perceived barriers to ADR reporting are illustrated in Figure 1, and further details regarding barriers are highlighted in Table 5.

### 3.6. Associations Between Demographic Characteristics and PV Aspects

**Figure 2:**
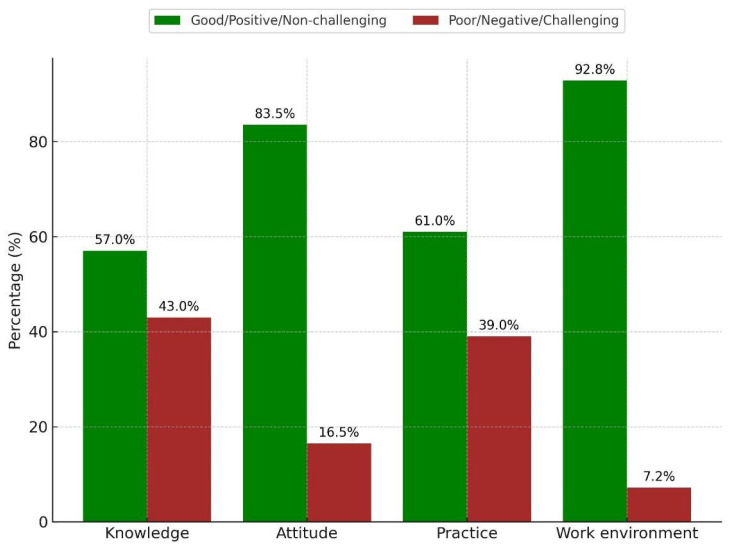
The distribution of knowledge (good vs. poor), attitude (positive vs. negative), practice (good vs. poor), and work environment (challenging vs. non-challenging) among study participants.

**Figure 3:**
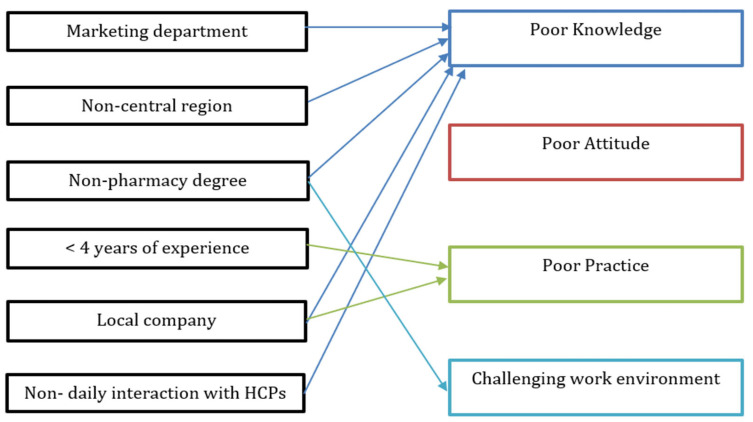
Conceptual framework of the associations between baseline characteristics and PV-related aspects.

The distribution of knowledge, attitude, practice, and work environment (assessed through perceived barriers) among study participants is illustrated in Figure 2, and the associations between the subjects’ baseline characteristics and PV-related aspects are presented in Table 6 and Figure 3. Poor knowledge was significantly associated with employment in marketing departments, residence in non-central regions, holding a non-pharmacy degree, working for local companies, and not interacting with HCPs on a daily basis. Poor practice was significantly linked to having fewer than four years of professional experience and working for local companies. Having a challenging work environment was significantly associated with holding a non-pharmacy degree.

## 4. Discussion

To the best of our knowledge, this is the first study that has evaluated KAP and barriers toward PV among pharmaceutical sales and marketing personnel. Our findings demonstrate that 57% and 83.5% of respondents in Saudi Arabia had good knowledge and positive attitude, respectively. The work environment was considered non-challenging by 92.8% of participants, and 61% reported good practice. We noted that holding a non-pharmacy degree was a significant predictor of poor knowledge and a challenging work environment, and working for local companies was significantly associated with poor knowledge and practice. Additionally, employment in marketing departments, residence in non-central regions, and not interacting with HCPs on a daily basis were significantly linked to poor knowledge, and having fewer than four years of professional experience was significantly linked to poor practice.

In the assessment of knowledge, the study findings were consistent with those reported by Abdulsalim et al. who reported good knowledge among pharmacists, who represent the majority of this study participants (97.8%) [11]. However, in both studies a considerable proportion of respondents were unable to identify the correct definition of PV and did not know the primary purpose of PV activities. This gap was more pronounced in our study, which could be attributed to differences in target sample sizes between the two studies and could be mitigated by encouraging QPPVs to better highlight their role by providing adequate training as mandated by the SFDA [10]. The association between poor knowledge and holding a non-pharmacy degree noted in our study supports the decision of limiting both sales and marketing roles to pharmacists. Additionally, the association between poor knowledge and employment in a local company could be attributed to comparatively limited pharmacovigilance infrastructure and fewer structured training opportunities. Lastly, given the association between working in non-central regions and poor knowledge, more intensive training for personnel in these regions is suggested.

Most of the existing literature supports the positive attitude toward PV observed in our study [11,16,17]. This trend is reflected globally as well as nearly all respondents (97%) in an Australian study conducted by Li and colleagues who surveyed 232 community pharmacists believed that ADR reporting is important and 93% felt that they are obliged to report ADRs [18]. In our study, only 51.8% of participants deemed the ADR reporting form not complex to fill out. While this may not accurately reflect the overall perception since 16% were unsure, likely due to a lack of prior experience with the form, this remains concerning. It can be tackled by urging QPPVs to offer personalized training to those who find the form complex. The training should emphasize what actually needs to be reported for a valid ADR report: a single patient information (initials, sex, weight, age or date of birth), suspected medicine, suspected adverse reaction, and an identifiable reporter (name, initials, address, contact information, or qualification) [19].

Despite that 61% of participants in our study were found to demonstrate good practice, more than one-third of respondents (39.8%) reported not having ever reported an ADR, and more than half (59.3%) reported not having reported an ADR in the last 12 months. These findings were substantiated by other studies. For example, 77% of community pharmacists and 73.2% of hospital pharmacists reported not having ever reported an ADR in Saudi Arabia and Kuwait, respectively [11,16]. This also mirrors the situation in the US, where 51% of HCPs reported not having submitted an ADR in the past five years [20]. Several strategies may help address this issue. For instance, enhanced awareness is needed to emphasize that even common and non-severe ADRs should be reported. This is imperative as a systematic review of 45 studies identified the belief that only severe ADRs need to be reported as the leading factor contributing to under-reporting [21]. Also, MAHs are encouraged to streamline the process by adopting PV systems that make ADR reporting straightforward. This is particularly important as a systematic review and meta-analysis identified integrating an electronic reporting manager as the most effective method to enhance ADR reporting and resulted in increasing reporting rate by more than five times [22]. Additionally, the association between good practice and multinational companies we observed in our study may possibly reflect the superior PV systems multinational companies utilize. Lastly, it is recommended that QPPVs exercise stricter oversight of sales and marketing personnel’s adherence to reporting practices as enhanced compliance is anticipated to positively influence overall reporting quality, and institutions are encouraged to acknowledge and recognize individuals with strong reporting records to foster a culture of proactive PV.

Regarding barriers, while most of the respondents in our study reported having non-challenging work environments (92.8%), a significant proportion indicated key obstacles to ADR reporting. Nearly half (44.8%) and (47%) felt they lack sufficient time to complete ADR reports and were not aware of the national ADR reporting system and how to use it, respectively. These findings were supported by other studies. For instance, Abdulsalim and colleagues reported that 41% felt they lack both the time and knowledge required for ADR reporting [11]. Additionally, Alsaleh et al. identified lack of knowledge as the most common barrier hindering pharmacists from ADR reporting in Kuwait (68.9%) [17]. These gaps can be addressed by targeted educational interventions. Also, MAHs are encouraged to reduce the workload of sales and marketing personnel to allow time for ADR reporting to be incorporated into their schedules. Another notable barrier is fear of professional repercussions. In our study, over one-third (38%) expressed concern that reporting ADRs could negatively impact their career progression or job security. Similarly, 69.3% of pharmacists in Najran, Saudi Arabia expressed concern regarding legal liability. These findings are alarming and warrant diligent attention by decision makers, including the SFDA. Given the high level of trust HCPs place in the SFDA, direct communication and reassurance from their end are anticipated to be impactful and influential in addressing this issue. Lastly, the highly positive attitude observed in our study did not translate into reporting practices. This suggests that barriers, particularly lack of awareness of the national ADR system, time constraints, and concerns regarding career progression or job security, play a critical role.

The National Pharmacovigilance Center (NPC) at the SFDA conducts regular inspections of the MAHs to assess their compliance with the PV-related regulations and guidelines. They select MAHs for inspection based on a risk-based methodology, which factors in various variables, including but not limited to the complexity of the organization and its PV system, the MAH reporting rate, its compliance and inspection history, as well as whether they have newly marketed products. Between 1 January 2025 and 30 June 2025, the NPC conducted 21 inspections and observed 87 findings classified as follows: seven critical, 55 major, and 25 minor findings. The critical findings were related to QPPV (*n* = 2), PSMF (*n* = 2), written instructions like SOPs and manuals (*n* = 1), management and reporting of adverse reactions (*n* = 1), and archiving (*n* = 1). Overall, considering the three categories, most of the findings were related to QPPV (*n* = 18), followed by PSMF (*n* = 15) and management and reporting of adverse reactions (*n* = 12). This trend has persisted throughout the past several years, with QPPV, PSMF, and management and reporting of adverse reactions being on the top of the findings list [23,24,25]. Given that these are integral parts of the QPPVs’ responsibilities, this highlights the significant role QPPVs play in this realm. Furthermore, it implies that MAHs should continue to work on fostering supportive environments that promote QPPVs’ continuous professional development as well as collaboration of other personnel with them.

The present study has some limitations. First, owing to that this is a self-administered, cross-sectional, survey-based study, our findings might have been impacted by a social desirability bias (i.e., the tendency of respondents to answer socially acceptable answers rather than those that reflect their true feelings and practices); hence, this limits the generalizability [26]. Second, given that there are no publicly available statistics regarding the number of pharmaceutical sales and marketing personnel in Saudi Arabia, we factored in a population size of 20,000 for the sample size calculation as suggested by the Roasoft calculator [14]. This population size seems reasonable based on internal industry estimates. Additionally, our use of email networks and various social media platforms to maximize reach among pharmaceutical sales and marketing personnel in Saudi Arabia. Moreover, our sample encompassed participants from diverse regions, companies (multinational and local), as well as experience levels, which have likely minimized the negative impact of this assumption on the reliability of our findings. Nonetheless, the lack of a precise population size may still limit the generalizability of our conclusions. Third, while multivariable regression analyses reflect the associations between the PV aspects and the subjects’ baseline characteristics more precisely, our study was only powered to use bivariate models. Four, only 2.3% of our participants had no pharmacy background, limiting the generalizability of our findings to this population in other countries, where pharmaceutical sales and marketing roles are not restricted to pharmacists. Lastly, internet penetration is nearly universal in Saudi Arabia (99%) and mobile access is almost ubiquitous [27]. Additionally, pharmaceutical companies in Saudi Arabia rely extensively on digital technologies and online communication platforms such as Teams, WhatsApp, and email groups to coordinate with their sales and marketing personnel, as well as our utilization of various online platforms likely have mitigated the risk of selection bias. Nevertheless, given that we relied on online dissemination of the questionnaire, the potential for selection bias cannot be entirely excluded.

## 5. Conclusions

Adverse effects and drug-related problems impose a significant burden on the healthcare system, and pharmaceutical sales and marketing personnel are among the most knowledgeable individuals regarding the medications they promote. Pharmaceutical sales and marketing personnel in Saudi Arabia demonstrated acceptable levels of KAP and reported few barriers toward PV, with an opportunity for improvement. This is further emphasized regarding their knowledge, particularly among those who hold a non-pharmacy degree and those who work in a local company, as well as their practice, particularly among those who have fewer than four years of professional experience and those who work in a local company. Strategies to address these deficiencies include: QPPVs are encouraged to provide personalized training and targeted educational interventions for personnel as mandated by the SFDA, MAHs are also encouraged to foster supportive environments that promote QPPVs’ continuous professional development as well as collaboration of other personnel with them, and the SFDA are suggested to reassure HCPs that reporting ADRs neither jeopardizes job security nor incurs legal liability.

## Figures and Tables

**Table 1:** Participants’ demographic data and work-related characteristics (*n* = 400).

	N	N%
**Current Position**		
In the sales department	377	94.3%
In the marketing department	23	5.8%
**Region**		
Eastern Region	72	18%
Central Region	229	57.3%
Western Region	58	14.5%
Northern Border Region	1	0.3%
Southern Region	40	10%
Age Group (years)		
18–24	6	1.5%
25–34	370	92.5%
35–44	22	5.5%
45–54	2	0.5%
**Gender**		
Male	229	57.3%
Female	171	42.8%
**Nationality**		
Saudi	394	98.5%
Non-Saudi	6	1.5%
**Academic Qualification**		
A degree in pharmacy (Bachelor, Diploma, ….)	391	97.8%
A degree in a related field (e.g., biology, chemistry, business administration)	9	2.3%
**Years of Experience in Pharmaceutical Field**		
Less than 2 years	74	18.5%
2–4 years	149	37.3%
4–6 years	121	30.3%
6–8 years	38	9.5%
More than 8 years	18	4.5%
**Type of Company**		
Multinational company	252	63%
Local company (including Arab companies) + Distributor (Agent)	148	37%
**Frequency of Interaction with Healthcare Professionals (HCPs)**		
Daily or almost daily	344	86%
2–3 times per week	27	6.8%
1–2 times per week	13	3.3%
Less than once per week	7	1.8%
1–2 times per month	6	1.5%
Less than once per month	3	0.8%

**Table 2:** Participants’ responses to the knowledge section.

**What is Pharmacovigilance (PV)?**				
The science of monitoring adverse drug reactions (ADRs) occurring in a hospital			94	23.5%
The process of improving the safety of drugs			15	3.8%
**The detection, assessment, understanding, and prevention of adverse effects**			**176**	**44%**
The science of detecting the type and incidence of adverse drug reactions (ADRs) marketing			110	27.5%
None of the above			5	1.3%
**What is the primary purpose of pharmacovigilance activities?**				
To identify predisposing factors to adverse drug reactions (ADRs)			59	14.8%
To identify unrecognized adverse drug reactions (ADRs)			98	24.5%
To calculate the incidence of adverse drug reactions (ADRs)			43	10.8%
**To enhance patient safety in relation to the use of drugs**			**195**	**48.8%**
None of the above			5	1.3%
**Which of the following best defines an adverse drug reaction (ADR)?**				
**Any noxious or undesired effect of a drug occurring at normal doses, during normal use**			**298**	**74.5%**
Adverse health outcomes associated with inappropriate drug use			35	8.8%
Harm resulting from the use of substandard/counterfeit drugs			22	5.5%
Harm caused by drug overdose			2	0.5%
Adverse outcomes associated with drug impurity			25	6.3%
Other health problems associated with drug use			18	4.5%
**Who may report ADRs at a pharmaceutical company?**				
Only Sales and Marketing team			19	4.8%
Only Pharmacovigilance team			54	13.5%
Only Medical team			9	2.3%
**Every single employee at the company**			**318**	**79.5%**
**Any adverse drug reaction (ADR) is considered serious if it:**				
Results in death			15	3.8%
Life-threatening			10	2.5%
Leads to hospitalization or prolongs existing hospitalization			15	3.8%
Causes significant disability/incapacity			2	0.5%
Leads to congenital anomaly (birth defect)			2	0.5%
Requires intervention to prevent permanent impairment or damage			5	1.3%
**All of the above**			**351**	**87.8%**
**Should only serious adverse drug reactions be reported?**				
Yes			68	17%
**No**			**321**	**80.3%**
Don’t Know			11	2.8%
**Should common and well-known adverse drug reaction (ADR) be reported?**				
**Yes**			**291**	**72.8%**
No			92	23%
Don’t Know			17	4.3%
**Which regulatory agency is responsible for pharmacovigilance activities in Saudi Arabia?**				
**National Pharmacovigilance Centre (NPC)**			**270**	**67.5%**
Saudi Commission for Health Specialties			50	12.5%
Ministry of Health (MOH)			80	20%
**Is the following information essential to include in an adverse drug reaction (ADR) report to make it valid (Identified Patient, Suspected drug, Description of the ADR, Reporter’s contact**				
**Yes**			**367**	**91.8%**
No			12	3%
Don’t Know			21	5.3%
**Knowledge groups**				
Good	228	57%		
Poor	172	43%		

**Table 3:** Participants’ responses to the attitude section.

**Is Adverse Drug Reactions (ADRs) Reporting Necessary for Patient Safety?**				
Yes			388	97%
No			9	2.3%
Don’t Know			3	0.8%
**Is Adverse drug reactions (ADRs) reporting a professional obligation for healthcare professionals?**				
Yes			350	87.5%
No			26	6.5%
Don’t Know			24	6%
**Is Adverse drug reactions (ADRs) reporting form complex to fill out?**				
Yes			129	32.3%
No			207	51.8%
Don’t Know			64	16%
**Do you think pharmacovigilance should be taught in detail to healthcare professionals?**				
Yes			324	81%
No			50	12.5%
Don’t Know			26	6.5%
**Attitude groups**				
Positive	334	83.5%		
Negative	66	16.5%		

**Table 4:** Participants’ responses to the practice section.

**Where do you Typically Report Adverse Drug Reactions (ADRs) in Your Organization?**				
Online reporting system			97	24.3%
Directly to the manager or supervisor			36	9%
To the Saudi Food and Drug Authority (SFDA)			39	9.8%
To the Ministry of Health (MOH)			6	1.5%
To the Regulatory Affairs department (RA)			16	4%
To the Pharmacovigilance team			206	51.5%
**Have you ever reported an Adverse Drug Reaction (ADR) to your company’s pharmacovigilance department?**				
Yes			241	60.3%
No			159	39.8%
**Have you reported an Adverse Drug Reaction (ADR) in the last 12 months?**				
Yes			163	40.8%
No			237	59.3%
**Are you familiar with the different sections of an Adverse Drug Reaction (ADR) report form (e.g., patient demographics, adverse event details, Drug information)?**				
Yes			314	78.5%
No			86	21.5%
**Have you received any training on pharmacovigilance principles and the use of ADR report forms?**				
Yes			358	89.5%
No			42	10.5%
**When do you typically report an Adverse Drug Reaction (ADR) in your organization?**				
Within 1 business day			267	66.8%
Within 5 business days			41	10.3%
At the end of the week			10	2.5%
At the end of the month			6	1.5%
No specific time frame			76	19%
**What is the first step you take when you have a valid Adverse Drug Reaction (ADR) report?**				
Contact the Pharmacovigilance team (via email, web, phone, etc.)			331	82.8%
Hand it over to your direct supervisor			46	11.5%
Contact the SFDA to submit the report			16	4%
Notify other HCPs about the ADR			7	1.8%
**Do you discuss Adverse Drug Reactions (ADRs) with healthcare professionals during your visits?**				
Yes			332	83%
No			68	17%
**Practice groups**				
Good	244	61%		
Poor	156	39%		

**Table 5:** Participants’ responses to the barriers section.

**Do you feel you lack sufficient time to complete ADR reports?**				
Yes			179	44.8%
No			221	55.3%
**Do you believe that ADR reporting is not part of your job role?**				
Yes			90	22.5%
No			310	77.5%
**Are you aware of the national ADR reporting system and how to use it?**				
Yes			212	53%
No			188	47%
**Have you received adequate training on ADR reporting?**				
Yes			309	77.3%
No			91	22.8%
**Do you feel confident in discussing ADRs with healthcare providers?**				
Yes			342	85.5%
No			58	14.5%
**Do you always have enough patient’s information to complete an ADR report?**				
Yes			189	47.3%
No			211	52.8%
**Do you always feel confident in determining whether an event is an ADR worth reporting?**				
Yes			298	74.5%
No			102	25.5%
**Do you feel your ADR reports make a significant difference in patient safety?**				
Yes			373	93.3%
No			27	6.8%
**Do you have concerns that your ADR reports could be incorrect or incomplete?**				
Yes			223	55.8%
No			177	44.3%
**Do you feel that reporting ADRs creates additional, unnecessary work for you?**				
Yes			130	32.5%
No			270	67.5%
**Do you believe that most ADRs are already well-documented before a drug is marketed?**				
Yes			215	53.8%
No			185	46.3%
**Is it always easy to contact the QPPV or relevant personnel for assistance with ADR reporting?**				
Yes			323	80.8%
No			77	19.3%
**Do you have concerns about patient privacy that may hinder your willingness to report ADRs?**				
Yes			207	51.8%
No			193	48.3%
**Does your company culture encourage open reporting of ADRs?**				
Yes			343	85.8%
No			57	14.3%
**Does your company provide adequate support and resources for ADR reporting?**				
Yes			335	83.8%
No			65	16.3%
**Are the ADR reporting systems and processes easy to use and understand?**				
Yes			340	85%
No			60	15%
**Do you feel that your manager or other supervisors encourage you to report ADRs?**				
Yes			313	78.3%
No			87	21.8%
**Do you feel that your career progression or job security could be negatively impacted by reporting ADRs?**				
Yes			152	38%
No			248	62%
**Barriers groups**				
Challenging	29	7.2%		
Non-challenging	371	92.8%		

**Table 6:** Associations between demographic characteristics and PV aspects.

	PV Aspect	Poor Knowledge	Poor Attitude	Poor Practice	Challenging Work Environment
Demographic					
**Marketing department**		0.009 *	1.00	0.082	0.075
**Non-central region**		0.001 *	1.00	0.097	0.334
**Non-pharmacy degree**		0.043 *	0.366	0.320	0.002 *
**<4 years of experience**		0.360	0.498	0.024 *	0.700
**Local company**		0.021 *	0.331	0.001 *	0.231
**Non-daily interaction with HCPs**		0.005 *	0.846	0.141	0.782

PV: pharmacovigilance; *: statistically significant.

## Data Availability

The original contributions presented in this study are included in the article/Supplementary Materials. Further inquiries can be directed to the corresponding author.

## Supplementary Materials

The following supporting information can be downloaded at: https://www.mdpi.com/article/10.3390/pharmacy13050145/s1, File S1: The study questionnaire.
