# Supplementary material for: Knowledge, Attitude, Practice, and Barriers Toward Pharmacovigilance Among Pharmaceutical Sales and Marketing Personnel in Saudi Arabia: A Cross-Sectional Study
Source: Pharmacy (Basel). 2025 Oct 9;13(5):145. doi: 10.3390/pharmacy13050145 (PMC12567404; doi:10.3390/pharmacy13050145)
Supplement: Supplementary file 1 [file pharmacy-13-00145-s001.zip › pharmacy-3838528-supplementary.pdf]

Supplementary File S1: The study questionnaire

A. Baseline Characteristics:

1. **Are you currently employed in a Marketing or Sales role within a pharmaceutical company in Saudi Arabia?**
   Within Sales department
   Within Marketing Department
   None (end of survey)

2. **In which region of Saudi Arabia do you currently work?**
   Central Region
   Eastern Region
   Western Region
   Northern Region
   Southern Region

3. **What is your age in years?**
   18-24
   25-34
   35-44
   45-54
   55-64
   More than 65

4. **What is your gender?**
   Male
   Female

5. **What is your nationality:**
   Saudi
   Non-Saudi

6. **What is your basic educational Qualification?**
   Degree in pharmacy (Bachelor, Diploma, ….)
   Degree in a relevant field (e.g., biology, chemistry, business administration)
   Other: _______________

7. **Professional experience in Marketing and sales?**
   less than 2 years
   2-4 years
   4-6 years
   6-8 years
   More than 8 years

8. **What is your company type?**
   Multinational company
   Local company (including Arab companies)
   Distributor (Agent)
   Other

9. **How often do you interact with Healthcare Professionals (HCPs)?**
Daily or almost daily
2-3 times per week
1-2 times per week
Less than once per week
1-2 times per month
Less than once per month

B. Knowledge:

1. **What is pharmacovigilance (PV)?**
The science of monitoring ADRs occurring in a hospital
The process of improving the safety of drugs
The detection, assessment, understanding, and prevention of adverse effects
The science of detecting the type and incidence of ADRs post-marketing.
None of the above

2. **What is the primary purpose of pharmacovigilance activities?**
To identify predisposing factors to ADRs
To identify unrecognized ADRs
To calculate the incidence of ADRs
To enhance patient safety in relation to the use of drugs
None of the above

3. **Which of the following best defines an adverse drug reaction (ADR)?**
Any noxious or undesired effect of a drug occurring at normal doses, during normal use
Adverse health outcomes associated with inappropriate drug use
Harm resulting from the use of substandard/counterfeit drugs.
Harm caused by drug overdose
Adverse outcomes associated with drug impurity
Other health problems associated with drug use

4. **Who may report ADRs at a pharmaceutical company?**
Sales and Marketing team
Pharmacovigilance team
Medical team
Every single employee at the company

5. **An ADR is considered serious if it:**
Results in death
Is life-threatening
Leads to hospitalization or prolongs existing hospitalization
Causes significant disability/incapacity
Leads to congenital anomaly
Requires intervention to prevent permanent impairment or damage
All of the above

6. **Are only serious ADRs required to be reported?**
   Yes
   No
   Don't know
7. **Should common and well-known ADRs be reported?**
   Yes
   No
   Don't know
8. **What is the regulatory agency responsible for pharmacovigilance activities in Saudi Arabia?**
   National Pharmacovigilance and Drug Safety Center (NPC)
   Saudi Commission for Health Specialties
   Ministry of Health (MOH)
9. **Is the following information essential to include in an ADR report to make it valid (identified patient, suspected drug, description of the ADR, reporter's contact information)?**
   Yes
   No
   Don't know

C: Attitude:

1. **Is ADR reporting necessary for patient safety?**
   Yes
   No
   Don't know
2. **Is ADR reporting a professional obligation for healthcare professionals?**
   Yes
   No
   Don't know
3. **Is the ADR reporting form complex to fill out?**
   Yes
   No
   Don't know
4. **Do you think pharmacovigilance should be taught in detail to healthcare professionals?**
   Yes
   No
   Don't know

D. Practice:

1. **Where do you typically submit ADR reports in your institution?**
   Web-based reporting system
   Directly to the manager or supervisor
   To the Saudi Food and Drug Authority (SFDA)
   To the Ministry of Health (MOH)
   To the Regulatory Affairs (RA) department
   To the Pharmacovigilance (PV) team

2. **Have you ever reported an Adverse Drug Reaction (ADR) to your company's pharmacovigilance department?**
   Yes
   No

3. **Have you reported an ADR in the last 12 months?**
   Yes
   No

4. **Are you familiar with the different sections of an ADR report form (e.g., patient demographics, adverse event details, drug information)?**
   Yes
   No

5. **Have you received any training on pharmacovigilance principles and the use of ADR report forms?**
   Yes
   No

6. **When do you typically report an ADR in your institution?**
   Within 1 business day
   Within 5 business days
   At the end of the week
   At the end of the month
   No specific time frame

7. **What is the first step you take when you have a valid ADR report?**
   Contact the Pharmacovigilance team (via email, web, phone, etc.)
   Hand it over to your direct supervisor
   Contact the SFDA to submit the report
   Notify other HCPs about the ADR

8. **Do you discuss ADRs with healthcare professionals during your visits?**
   Yes
   No

E. Barriers:

1. **Do you feel you lack sufficient time to complete ADR reports?**
   Yes
   No
2. **Do you believe that ADR reporting is not part of your job role?**
   Yes
   No
3. **Are you aware of the national ADR reporting system and how to use it?**
   Yes
   No
4. **Have you received adequate training on ADR reporting?**
   Yes
   No
5. **Do you feel confident in discussing ADRs with healthcare providers?**
   Yes
   No
6. **Do you always have enough information from patients to complete an ADR report?**
   Yes
   No
7. **Do you always feel confident in determining whether an event is an ADR worth reporting?**
   Yes
   No
8. **Do you feel your ADR reports make a significant difference in patient safety?**
   Yes
   No
9. **Do you have concerns that your ADR reports could be incorrect or incomplete?**
   Yes
   No
10. **Do you feel that reporting ADRs creates additional, unnecessary work for you?**
    Yes
    No
11. **Do you believe that most ADRs are already well-documented before a drug is marketed?**
    Yes
    No
12. **Is it always easy to contact the QPPV or other relevant personnel for assistance with ADR reporting?**
    Yes
    No

13. **Do you have concerns about patient privacy that may hinder your willingness to report ADRs?**
    Yes
    No
14. **Does your company culture encourage open reporting of ADRs?**
    Yes
    No
15. **Does your company provide adequate support and resources for ADR reporting?**
    Yes
    No
16. **Are the ADR reporting systems and processes easy to use and understand?**
    Yes
    No
17. **Do you feel that your manager or other supervisors encourage you to report ADRs?**
    Yes
    No
18. **Do you feel that your career progression or job security could be negatively impacted by reporting ADRs?**
    Yes
    No
